# Why authors of research papers must provide evidence of ethical research practice

**DOI:** 10.1016/j.heliyon.2023.e20476

**Published:** 2023-09-29

**Authors:** Simon E. Kolstoe

**Affiliations:** University of Portsmouth, Portsmouth, UK

## 1 Introduction

In a recent editorial [1] Cornock and Rees describe the ethics requirements necessary for academic publication. Briefly these are i) review by a Research Ethics Committee (REC) (or an equivalent Institutional Review Board (IRB)), and ii) evidence of participant consent. They rightly base their requirements on international documents such as the Nuremberg Code [2], Declaration of Helsinki [3], and the Belmont report [4]. The Belmont report in particular is helpful because it goes on to introduce the specific ethics principles that must be considered when conducting research. Journals play an important role by providing a platform that authors can use to demonstrate that these ethics principles have been met.

So what are these principless, and why are they important for “good research”? It is not well known that one of the young staff officers responsible for writing the 1978 Belmont report was Tom Beauchamp, who later authored the textbook “*Principles of Biomedical Ethics*” with James Childress [5], that was influential in cementing the “Principlist” approach to ethics within medicine, and applied ethical considerations, more generally. The most common form of this theory emphasises respect for the person (autonomy), the principle of beneficence, and the principle of justice (all three originally outlined in the Belmont report) alongside a fourth principle of non-maleficence (do no harm). Principlism has proven useful to clinicians, authors, researchers and indeed publishers as it provides an accessible framework for dealing with immediate ethical queries without having to refer to more complex, normative, philosophical theory (such as deontology, consequentialism, virtue ethics etc.). It also provides an element of common ground in the approach to ethics across medicine, research and other applied activities where ethics decisions need to be made rapidly or in the heat of the moment.

However, while Principlism is indeed a helpful guide for many practitioners including researchers, the complexity of research often sees the headline principles interpreted in different ways when guiding day to day activities. For instance “beneficence” is most commonly interpreted as considering the overall scientific and social benefits of research, which encompasses ideas relating to transparency, open data, reproducibility, and reliable research practice. Similarly research misconduct, fraud, plagiarism, falsification and fabrication are all issues related to non-maleficence, while issues linked to consent procedures and participant information relate to the principle of autonomy. Justice is also important within research, perhaps particularly emphasised by recent feminist [6] and de-colonial critiques [7] of research priorities and culture. As a consequence many of the issues that relate to research culture, integrity, and generally just good research behaviour, have their foundation in the four principle approach to ethics.

Journals asking for proof that authors have addressed these ethical principles are therefore not just making requests for the sake of it. Rather than being an administrative hoop, or seeking to “tick the box” that researchers have received some form of committee ethics review, or got their research participants to sign a particular piece of paper or form, they are instead a specific request for proof that researchers have engaged with, and are demonstrating, good research practice itself. Not being able to produce such evidence is essentially research misconduct, and along with being justification for the journal not publishing an article, could also be reported back to researcher's employers as a research integrity or misconduct issue.

## 2 Poor ethics is poor science

Research is an incredibly important and fruitful activity, so those seeking to contribute to research knowledge must be held to high standards of conduct. If authors submitting manuscripts to journals cannot provide evidence that they have engaged with ethical principles, their research is essentially useless because there are no reassurances that the work is reliable or to be trusted. This is a matter of research culture, practice and environment, where activities or knowledge seeking to be called “research” must demonstrate these higher standards of moral and social accountability so as to distinguish research from other forms of knowledge such as journalism, anecdote or hearsay. Even related forms of knowledge such as “market research”, or education/teaching, fall short of the standard required for high quality research. For instance consider how much medical education is based on personal experience passed on between clinicians as opposed to robust research derived evidence. It is therefore a journals responsibility to ensure that all work published in its pages claiming to be research has met these high standards, and likewise the institutions funding and conducting the research must ensure that anything presented to a journal is assessed against the appropriate standards.

Over recent years there has been an increased interest in the ethical conduct of research. The COVID-19 pandemic in particular emphasised the transformative effects that good research and researchers can have when focussed on finding solutions to worldwide problems. However, at the same time poor research practice such as, for instance, the fraud relating to previous vaccine research [8] can significantly damage public trust, and indeed cost lives. High profile retractions such as those that led to the recent resignation of the University of Stanford's president [9] can cause many politicians and members of the public to question the privileged position of research and scientifically derived knowledge [10]. It is therefore the responsibility of everyone involved with research to uphold standards and behaviours. Science is a human activity and as such is far from perfect. Questionable research practices are common, and sit on a spectrum from honest mistakes through to harmful, and sometimes deadly, fraud. This is a topic that must be taken seriously by researchers and journal editors alike. Proof of ethics review, and proof of participant consent in particular, is therefore essential for journals to reassure their readers that the work in their pages is genuine, ethical, and is providing information that can be trusted.

## CRediT authorship contribution statement

**Simon Kolstoe:** Conceptualization, Writing - original draft, Writing - review & editing.

## Declaration of competing interest

The authors declare that they have no known competing financial interests or personal relationships that could have appeared to influence the work reported in this paper.
